# Bromide of Potash and the Liver

**Published:** 1892-05

**Authors:** 


### BROMIDE OF POTASH AND THE LIVER.

A large share of the patent medicines advertised in the newspapers and offered for sale in drug stores as "nervines and sleep producers" contain, as their principal ingredient, either opium or bromide of potash ; the least harmful owe their efficiency to the last named agent. The temporary benefit derived from these nostrums leads many people to become habitual users of them, taking bottle after bottle of somebody's "nervine" or "sleep producer," with no appreciation of the possible harm which may arise from the long continued use of cheap but powerful drugs which the nostrum may contain; and their half paralyzing influence in lessening the activity of the brain, weakening the memory, and lowering the nerve tone to such a degree as to produce unsteadiness of gait, is known. It has recently been shown by MM. Hare and Herber, of Paris, that when this drug is administered for a long time, it accumulates in the liver so that its poisonous principles are concentrated upon this organ.

This explains how the habitual use of one nostrum creates a demand for another. The long continued use of a medicine containing bromide of potash, after a while renders the liver so inactive that somebody's patent "liver regulator" must be resorted to to stimulate the enfeebled organ. Thus one mischief leads to another.—*Good Health.*

